# Parental Threats and Anxiety Severity in Trauma-Exposed Adolescents: The Role of Anxiety Sensitivity

**DOI:** 10.1007/s40653-026-00938-8

**Published:** 2026-07-01

**Authors:** Dania Y. Amarneh, Mallory R. Cotton, Emily L. Mallin, Andres G. Viana

**Affiliations:** 1 Department of Psychology, University of Houston, Houston, USA; 2 Department of Psychological and Brain Sciences, Texas A&M University, College Station, USA

**Keywords:** Parental threat, Anxiety, Trauma, Anxiety sensitivity, Adolescents

## Abstract

Introduction: The present investigation examined associations between parental threatening behaviors (i.e., threats of rejection, abandonment, and/or punishment), anxiety sensitivity (i.e., the belief that physiological sensations of anxiety are dangerous and signal imminent catastrophe), and anxiety symptom severity in a diverse sample of trauma-exposed adolescents in acute psychiatric inpatient care. We hypothesized that greater exposure to parental threatening behaviors would be associated with more severe anxiety symptoms via heightened anxiety sensitivity. Method: Participants were 50 trauma-exposed adolescents ages 12 to 17 years (52.0% female; *M*_*age*_ = 15.1 years; 56% ethnic minority) in inpatient care who completed measures of parental threatening behaviors, anxiety sensitivity, and anxiety symptom severity. Results: Statistically significant indirect effects were found between parental threatening behaviors and anxiety symptom severity through anxiety sensitivity (ab: *β* = 0.15, *SE* = 0.06, 95%CI [0.03, 0.28]). Post-hoc exploratory analyses of the anxiety sensitivity facets revealed similar findings. Conclusion: Anxiety sensitivity partially explained the association between parental threatening behaviors and anxiety symptom severity in a trauma-exposed sample of adolescents in psychiatric inpatient care. Findings revealed that parental threatening behaviors are associated with anxiety psychopathology in this population and demonstrate the potential utility of evaluating anxiety sensitivity-focused interventions for this population.

Anxiety and its disorders are highly prevalent among adolescents (ages 12–17 years; Sappenfield et al., 2024; Silva et al., 2020). Recent epidemiological work in the United States places the prevalence of anxiety disorders in this age group at 20%, more than twice the rate of depression in the same age group (8%; Sappenfield et al., 2024). Among those who have experienced a traumatic event (Stangeland et al., 2024) and/or are receiving psychiatric inpatient care (Mainali et al., 2023), prevalence rates can be considerably higher. Given its association with increased psychiatric comorbidity, academic disengagement, psychosocial dysfunction, and even suicide (Copeland et al., 2014; Sappenfield et al., 2024), anxiety psychopathology in adolescence represents a significant public health concern (Yang et al., 2021). Yet, scientific knowledge of the parental and cognitive mechanisms that contribute to anxiety symptom severity, both in general and among psychiatric inpatient populations (Zambrowicz et al., 2019), remains limited.

## Parental Threatening Behaviors and Anxiety Severity in Adolescence

Attachment theory posits that children develop internal working models, or mental representations of themselves and others, based in part on caregivers’ responsiveness and availability (Bowlby, 1969, 1973). Consistent, sensitive caregiving fosters a secure attachment, which is characterized by comfort with emotional closeness, trust in the caregiver’s availability, and confidence in using the caregiver as a secure base from which to explore the environment (Ainsworth et al., 2015; Mikulincer et al., 2003). In contrast, inconsistent, neglectful, or threatening caregiving increases the likelihood of developing an insecure or anxious attachment, characterized by uncertainty about the caregiver’s availability, discomfort or anxiety in seeking support, and difficulties regulating emotions (Ainsworth et al., 2015; Mikulincer et al., 2003). Unsurprisingly, insecure attachment is a significant predictor of anxiety disorders (see (Kobak et al., 2006) for a review).

Within this developmental framework of parent-child relationships, Bowlby (1980) argued that *parental threatening behaviors*—such as threats of rejection, threats of abandonment, and threats of punishment—are particularly salient predictors of youths’ future maladaptive psychosocial functioning (Bowlby, 1969; Clayborne et al., 2021; Lei et al., 2023; Rasing et al., 2020; Scher et al., 2002). Threats of rejection include behaviors that suggest emotional unavailability by the caregiver, while threats of abandonment are represented by behaviors that suggest a lack of physical availability. Threats of punishment involve behaviors implying that the caregiver may physically hurt the adolescent or an individual important to the adolescent. These profoundly destabilizing threats, especially in the context of chronic exposure to such behaviors within the primary caregiving relationship, are thought to disrupt the formation of secure attachment bonds, amplify fears of uncertainty in adolescents, and elicit concerns in youth for their safety and well-being.

Compelling evidence suggests that parental threatening behaviors increase risk for anxiety-related psychopathology among adolescents (Clayborne et al., 2021; Lei et al., 2023; McLeod et al., 2007). In a community sample, harsh parenting (a parenting style closely associated with parental threats) was both directly and indirectly (through interpersonal sensitivity and ego-depletion) associated with elevated anxiety symptoms in adolescents (Li et al., 2025). One meta-analysis also reported statistically significant associations between negative parenting behaviors and family conflict and social anxiety in youth (Lei et al., 2023). Significant associations between parental threatening, rejecting, and hostile behaviors and current anxiety symptoms were also found in a sample of late adolescents and young adults (Scher & Stein, 2003). Although these findings highlight the role of parental threatening (and related harsh/negative) behaviors in the development of anxiety psychopathology among adolescents, the specific cognitive-affective mechanisms involved (e.g., Scher & Stein, 2003) remain understudied. Identifying such mechanisms could inform efforts to implement and disseminate brief evidence-based practices to inpatient settings, where resources and time are limited (Gupta et al., 2024; Raphael et al., 2021).

### Anxiety Sensitivity

One candidate mechanism linking parental threatening behaviors to anxiety symptomatology is anxiety sensitivity (Reiss, 1991; Reiss et al., 1986; Taylor et al., 2007). Anxiety sensitivity is defined as the fear of anxiety-related sensations due to the belief that they signal impending catastrophe (Reiss, 1991; Reiss et al., 1986; Taylor et al., 2007). An adolescent with high anxiety sensitivity may interpret an increase in heart rate as an impending heart attack, or view perspiration as cause for social rejection. Anxiety sensitivity in youth is comprised of four facets (Silverman et al., 2003): mental incapacitation concerns (e.g., “When I am afraid, I fear I may be going crazy”), social concerns (e.g., “I don’t want others to know when I’m afraid”), disease concerns (e.g., “It scares me when my heart beats fast”), and unsteady concerns (e.g., “It scares me when I have trouble catching my breath”). Associations between heightened anxiety sensitivity and anxiety symptom severity have been reported in samples of youth (Hernandez Rodriguez et al., 2020; Knapp et al., 2016; Naragon-Gainey, 2010; Weems et al., 2002), including those in acute psychiatric care (Amarneh et al., 2022). Anxiety sensitivity also predicted internalizing symptoms (including anxiety) among fourth-grade children across an academic year (Hernandez Rodriguez et al., 2020). Notably, anxiety sensitivity is especially elevated among trauma-exposed youth (Chiu et al., 2024; McLaughlin & Hatzenbuehler, 2009), contributing to greater internalizing psychopathology in this population (Chiu et al., 2024).

Anxiety sensitivity is likewise associated with parental threatening behaviors (Erozkan, 2012; Scher & Stein, 2003; Viana & Stevens, 2016; Watt & Stewart, 2003). In a sample of high school students, parenting styles explained 29.1% of the variance in anxiety sensitivity (Erozkan, 2012), with negative/harsh parenting styles being positively associated with anxiety sensitivity, and democratic parenting negatively associated (Erozkan, 2012). Greater exposure to parental threatening behaviors was also associated with more severe anxiety sensitivity among young adults (Viana & Stevens, 2016). Moreover, in those with higher anxiety sensitivity, parental threatening behaviors were associated with more frequent illicit substance use (Viana & Stevens, 2016). Related work found that parent emotional availability—arguably the inverse of parental threatening behaviors—was negatively associated with adolescent anxiety, with anxiety sensitivity partially mediating this association (Kramer & Francis, 2025).

Yet, the underlying role of anxiety sensitivity in the association between parental threatening behaviors and anxiety symptoms among adolescents has not been examined. Parental threatening behaviors can theoretically disrupt a child’s sense of safety (Lei et al., 2023; Scher et al., 2002), making the child more likely to perceive bodily arousal as a sign of danger (i.e., anxiety sensitivity) rather than as a normative stress response. Repeated exposure to this negative caregiving style may heighten children’s vigilance toward internal sensations and reinforce catastrophic interpretations (Ehlers & Clark, 2000; McLaughlin et al., 2020). In this vein, child-reported family dysfunction (i.e., inconsistent, unpredictable family environment) was associated with anxious youths’ negative interpretations (Blossom et al., 2013). Such patterns may be especially pronounced in trauma-exposed youth, who often display heightened physiological and cognitive vigilance as an adaptation to unsafe, threatening environments (Machlin et al., 2024; McLaughlin et al., 2019, 2020).

### The Present Study

The present investigation examined the underlying role of anxiety sensitivity in the association between parental threatening behaviors and anxiety symptom severity in a sample of trauma-exposed adolescents in psychiatric inpatient care. Based on previous work (Knapp et al., 2016; Viana & Stevens, 2016), the present investigation hypothesized that (a) parental threatening behaviors would be positively associated with anxiety sensitivity, (b) anxiety sensitivity would be positively associated with anxiety symptom severity, and (c) parental threatening behaviors would be positively associated with anxiety symptom severity. It was further expected that (d) parental threatening behaviors would be indirectly associated with anxiety symptom severity through elevated levels of anxiety sensitivity. Age, sex, and race were evaluated as covariates given higher rates of internalizing symptoms in girls and marginalized racial/ethnic minorities, as well as differences across age groups (Li et al., 2023; Nivard et al., 2017; Rasing et al., 2020; Silva et al., 2020).

## Method

### Participants

The sample of participants used in the present study was obtained from a previously conducted parent study examining individual differences in cognitive-affective vulnerabilities for psychopathology among youth admitted for acute psychiatric inpatient care at a state hospital (Viana et al., 2018). The original sample consisted of 74 adolescents aged 12 to 17 years. Potential participants were excluded if they reported: (a) active psychosis, (b) current suicidal or homicidal risk, (c) diagnosis of intellectual disability or autism spectrum disorder, or (d) a physical disability prohibiting the use of a computer. Study personnel were not involved in evaluating suicidality; the attending psychiatrist assessed suicide risk upon each adolescent’s hospital admission. If an adolescent was deemed to be actively suicidal by the attending psychiatrist during intake, they were not referred to the study for potential participation. The informed consent document also granted permission for the hospital to release the participants’ diagnoses and demographic information to the research staff, which allowed for assessment of remaining exclusion criteria.

Participants then completed a battery of self-report questionnaires, including the Childhood PTSD Symptoms Scale (CPSS; Foa et al., 2001). During completion of the CPSS, adolescents were asked to report the “most distressing event” they had experienced; only adolescents who reported exposure to a potentially traumatic event (i.e., a Criterion A event as defined by the PTSD diagnostic criteria of the *Diagnostic and Statistical Manual of Mental Disorders*,* 5th edition* [DSM-5; American Psychiatric Association, 2013 ]) were included in the present analysis. Twenty-four subjects were excluded from the current study for either: (1) not reporting a traumatic event on the CPSS; or (2) reporting an event that was not consistent with a Criterion A event for PTSD as outlined in the DSM-5 (e.g., breakup with boyfriend). The final sample was comprised of 50 trauma-exposed adolescents ages 12 to 17 years. Sample demographics are listed in Table 1.

**Table 1:** Sample demographics (*N* = 50)

Variable	Mean (SD) / n (%)
Sex	
Female	26 (52%)
Male	24 (48%)
Age	15.1 (1.4)
Race	
White/European American	22 (44%)
Black/African American	18 (36%)
Multiracial	10 (20%)
Primary diagnosis	
Mood disorder	27 (54%)
Attention deficit/hyperactivity disorder	9 (18%)
Disruptive behavior disorders	8 (16%)
Other psychiatric disorder	6 (12%)
Past suicide attempt	20 (40%)
Past suicidal ideation	21 (42%)
Current use of psychiatric medication	45 (90%)
Traumatic event endorsed	
Unexpected death of a loved one	16 (32%)
Sexual assault	14 (28%)
Physical assault	9 (18%)
Witnessing violence	4 (8%)
Other	4 (8%)
Life-threatening illness/ accidental injury	3 (6%)
Probable PTSD diagnosis	20 (40%)

*Note. Sex*,* Age*,* Race*, Demographics Questionnaire

*Primary diagnosis, Past suicide attempt, Past suicidal ideation, Current use of psychiatric medication,* *Medical Chart Review *

*Traumatic event endorsed, *Childhood PTSD Symptoms Scale (CPSS; Foa et al., 2001)

*Probable PTSD diagnosis, *total CPSS score of 16 or greater

### Measures

#### Parental Threatening Behaviors

##### ***Parent Threat Inventory****(****PTI;*** Scher et al., 2002)

The PTI is a 17-item self-report questionnaire that assesses experiences of parental threatening behaviors in childhood. These behaviors include (1) threats of rejection or unavailability (e.g., “My mother said she would stop taking care of me if I did not behave”), (2) threats of abandonment (e.g., “My mother threatened to divorce my dad”), and (3) threats of punishment or neglect (e.g., “My mother raised her hand as if she was going to hit me”). Adolescents indicated on a 5-point Likert scale from 1 (*never true*) to 5 (*very often true*) how often they experienced certain parental threatening behaviors, specifically from their mother, during their childhood. The measure yields a total score ranging from 17 to 58, with higher scores indicating greater exposure to parental threatening behaviors. Individual factor scores can be calculated by summing the score for items falling into each of the three categories of parental threatening behaviors (i.e., rejection, abandonment, punishment/neglect). Maternal threatening behaviors were examined because mothers (vs. fathers) play a significantly larger role in child-rearing in the U.S (AARP & National Alliance for Caregiving, 2015; Kotila et al., 2013; Pan et al., 2024). The PTI has consistently shown excellent validity and reliability in both emerging adult and adolescent samples (Scher et al., 2002; Trent et al., 2023; Viana & Stevens, 2016). In the present study, the PTI total score was examined as a predictor and demonstrated good internal consistency (α = 0.89).

#### Anxiety Sensitivity

##### ***Childhood Anxiety Sensitivity Index (CASI;*** Silverman et al., 1991, 2003)

The CASI is an 18-item self-report questionnaire that assesses the fear of anxiety-related sensations in children and adolescents. Adolescents were asked to rate the extent to which they experience fearful reactions to normal anxiety responses on a 3-point scale (1 = *none*, 2 = *some*, 3 = *a lot*). A total score for anxiety sensitivity was obtained by summing the responses to each item, with scores ranging from 18 to 54 (α = 0.90). Higher scores on the CASI are indicative of greater anxiety sensitivity. The measure also yields four subscales, which assess fear of anxiety-related sensations in the context of the following specific domains: disease concerns (e.g. “It scares me when my heart beats fast;” α = 0.84), social concerns (e.g. “I don’t want others to know when I’m afraid;” α = 0.51), mental incapacitation concerns (e.g. “When I am afraid, I fear I may be going crazy;” α = 0.66), and unsteady concerns (e.g. “It scares me when I have trouble catching my breath;” α = 0.72; Silverman et al., 2003). The CASI has demonstrated good validity and reliability in both clinical and non-clinical samples (Silverman et al., 1991). The four CASI subscales have also been examined among both clinical and nonclinical samples and have shown internal consistencies ranging from questionable to good (α = 0.62 to 0.83; Hair et al., 2010; Silverman et al., 2003). Goodness-of-fit indices support the use of the four-facet model of the CASI (Silverman et al., 2003).

### Anxiety Symptom Severity

#### ***Revised Children’s Manifest Anxiety Scale****(****RCMAS;*** Reynolds & Richmond, 1978***)***

The RCMAS is a 37-item self-report scale that assesses anxiety symptoms in youth. Adolescents responded to 28 items (*yes/no*) to indicate whether each item accurately described their feelings or actions. The measure yields a total score of anxiety symptom severity, as well as three subscale scores: physiological anxiety, worry/oversensitivity, and social concerns/concentration. The nine remaining items comprise the social desirability scale and are not included in the total anxiety score. Endorsed items were summed to yield a total anxiety score, which ranges from 0 to 28, with higher scores indicating greater anxiety symptom severity. The RCMAS has shown good validity and reliability (Turgeon & Chartrand, 2003). In this study, the internal consistency of the RCMAS was good (α = 0.82).

### Procedure

Adolescents admitted for acute psychiatric treatment at a state hospital were informed about the opportunity for participation in the study by trained study staff upon admission. Adolescents who provided assent and whose guardians provided informed consent were evaluated for inclusion in the study. Adolescents who met the inclusion criteria completed the study questionnaire battery within seven days of admission. During one study session lasting about 1.5 h, trained study staff administered a packet of questionnaires individually to each participant. All procedures were approved by the Institutional Review Boards (IRB) of both the state hospital where the data collection took place and the University of Mississippi Medical Center.

### Data Analytic Plan

Firstly, bivariate correlations, t-tests, and analyses of variance were conducted to examine main and interactive effects of demographic variables (i.e., sex, age, race) on parental threatening behaviors, anxiety symptom severity, and anxiety sensitivity. Second, tests of indirect effects were conducted to examine the association of parental threatening behaviors (PTI total score) on anxiety symptom severity (RCMAS total score) through anxiety sensitivity (CASI total score), using 10,000 bootstrap re-samplings via Model 4 of the PROCESS Macro for SPSS (Hayes, 2017). A bootstrap confidence interval (CI) that did not include zero provided evidence of a significant indirect effect (Preacher & Hayes, 2008).

## Results

### Preliminary Analyses

Two participants had missing data on the PTI total score; as such, mean imputation was used to replace missing values (Tabachnick & Fidell, 2007). No evidence of multicollinearity was identified between any study variables, and the distribution of all study variables approximated normality and demonstrated acceptable levels of skewness and kurtosis. A summary of bivariate correlations is presented in Table 2.

Age was significantly negatively associated with anxiety sensitivity *(r* = − .36; *p* = .010) and was thus included as a covariate. A one-way ANOVA revealed significant differences in child anxiety symptom severity as a function of race/ethnicity, (*F* [2, 47] = 4.75, *p* = .015). Tukey post-hoc comparisons revealed that White American children reported significantly higher anxiety symptoms (*M* = 16.59, *SD* = 8.09) than African American children (*M* = 9.56, *SD* = 6.83; *p* = .015). Race was therefore included as a covariate. Although *t*-tests revealed no significant sex differences among any of the main study variables, sex was retained as a covariate due differences in past work (Rasing et al., 2020; Silva et al., 2020). Positive and statistically significant associations were observed between parental threatening behaviors and anxiety sensitivity *(r* = .40; *p* = .004). Parental threatening behaviors *(r* = .35; *p* = .012) and anxiety sensitivity *(r* = .66; *p* < .001) were also statistically significantly associated with anxiety symptom severity.

**Table 2:** Bivariate correlations (*N* = 50)

	1	2	3	4	5	6	7	8
1. Age	-							
2. Parental threatening behaviors	-0.06	-						
3. Anxiety sensitivity	-0.36^**^	0.40^**^	-					
4. CASI disease concerns	-0.35^*^	0.41^**^	0.94^***^	-				
5. CASI unsteady concerns	-0.39^**^	0.25	0.85^***^	0.71^***^	-			
6. CASI mental incapacitation concerns	-0.34^*^	0.44^*^	0.83^***^	0.75^***^	0.64^***^	-		
7. CASI social concerns	-0.01	0.15	0.58^***^	0.41^**^	0.37^**^	0.36^*^	-	
8. Anxiety	-0.27	0.35^*^	0.66^***^	0.66^***^	0.54^***^	0.55^***^	0.30^*^	-
Mean	15.06	29.92	32.80	13.82	7.60	4.54	6.84	12.72
Standard Deviation	1.41	13.07	8.23	4.20	2.33	1.68	1.57	8.21
Range	12–17	17–71	21–53	8–23	4–12	3–9	3–9	0–28

*Note. Age*, Demographics Questionnaire; *Parental threatening behaviors*, Parental Threat Inventory (PTI); *Anxiety Sensitivity*,* Disease Concerns*,* Unsteady Concerns*,* Mental Incapacitation Concerns*,* Social Concerns*, Childhood Anxiety Sensitivity Index (CASI); *Anxiety*, Revised Children’s Manifest Anxiety Scale (RCMAS)

* *p* < .05; ** *p* < .01; *** *p* < .001

### Test of Indirect Effects

**Fig. 1:**
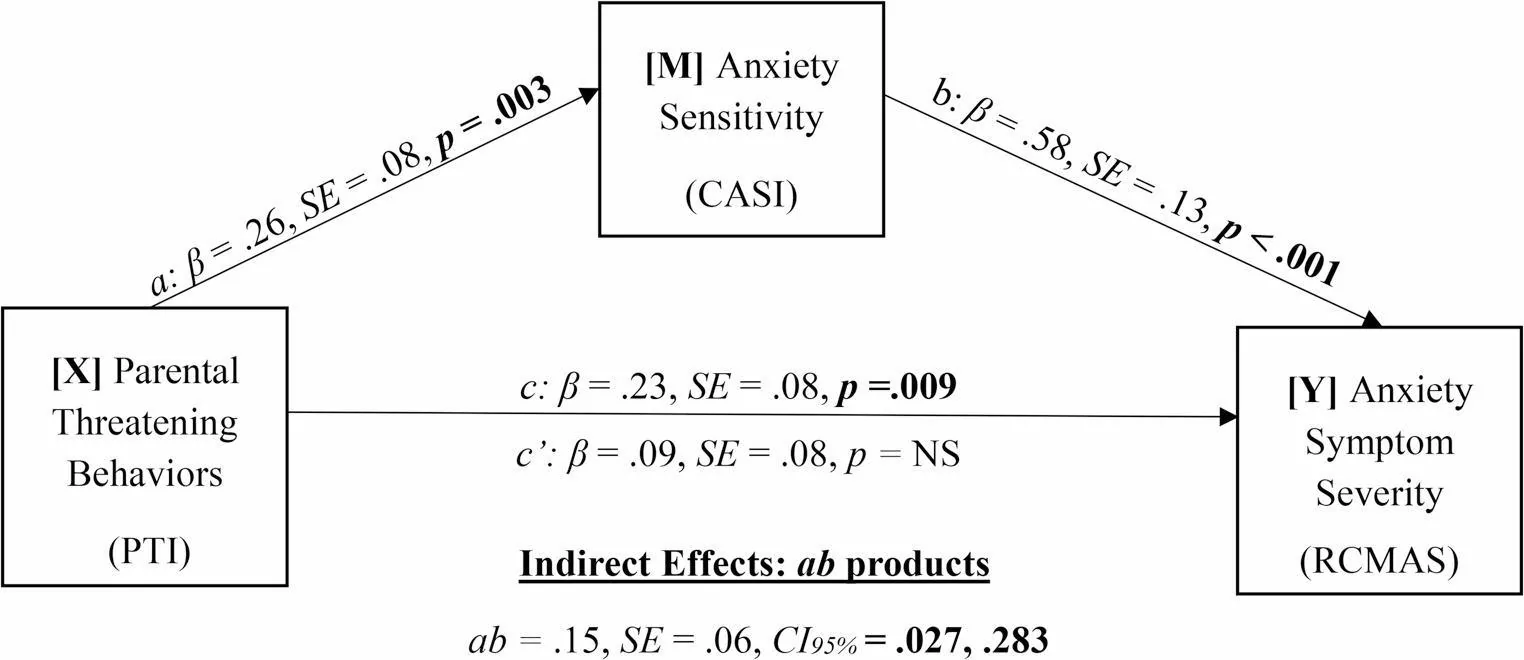
Main mediation model. Note. This figure depicts the main mediation model tested, whereby parental threatening behaviors (PTI total score) is associated with anxiety symptom severity (RCMAS total score) indirectly via anxiety sensitivity (CASI total score). Note: a, direct effect of X on M; b, direct effect of M on Y; c, total effect of X on Y; c′, direct effect of X on Y controlling for M; a*b, indirect effect of M

A summary of the results of indirect tests is illustrated in Fig. 1. Parental threatening behaviors were significantly associated with anxiety sensitivity (*a: β = .*26, *SE =* .08, *95% CI* [.09, .42]), and anxiety sensitivity was significantly associated with anxiety symptom severity (*b*: *β* = .58, *SE* = .13, *95% CI* [.31, .85]). The total effect of the model was statistically significant (*c: β* = .23, *SE* = .08, 95%CI [.06, .40]). The direct effect of parental threatening behaviors on anxiety symptom severity was not statistically significant (*c’*: *β* = 0.09, *SE* = 0.08, *95% CI* [–0.07, 0.25]). However, the indirect effect of parental threatening behaviors on anxiety symptom severity through anxiety sensitivity was statistically significant (*ab*: *β =* 0.15, *P*_*M*_ = 0.67, *SE* = 0.06, 95%CI [0.03, 0.28]).

### *Post hoc* Exploratory Analyses

A parallel mediation model (Kane & Ashbaugh, 2017) was tested to examine the effects of parental threatening behaviors on anxiety symptom severity through the four subscales of anxiety sensitivity: disease concerns, mental incapacitation concerns, unsteady concerns, and social concerns. A summary of results is illustrated in Table 3. Parental threatening behaviors were positively associated with both disease concerns (*a: β* = .13, *SE* = .04, *95% CI* [.05, .22]) and mental incapacitation concerns (*a: β* = .06, *SE* = .02, *95% CI* [.02, .09]), but not with unsteady concerns (*a: β* = .04, *SE* = .02, *95% CI* [–.00, .09]) or social concerns (*a: β* = .02, *SE* = .02, *95% CI* [–.02, .06]). Of the four subscales, only disease concerns were significantly related to greater anxiety symptom severity (*b*: *β* = .89, *SE* = .39, *95% CI* [.10, 1.69]). The total effect of parental threatening behaviors on anxiety symptoms was significant (*c: β* = .23, *SE* = .08, *95% CI* [.06, .40]). The direct effect of parental threatening behaviors on anxiety symptoms was not significant (*c’*: *β* = 0.08, *SE* = 0.08, *95% CI* [–0.09, 0.25]). However, the total indirect effect of the four subscales was significant (*ab*: *β* = 0.16, *P*_*M*_ = 0.67, *SE* = 0.07, *95% CI* [0.01, 0.29]).

**Table 3:** Standardized regression coefficients: parallel mediation model

Model	Model *R*^2^	β	SE	Bootstrapped 95% CI	
Parental threatening behaviors ➔ Disease concerns (*a*_1_)	0.30^**^	0.135	0.041	0.052	0.217
Parental threatening behaviors ➔ Unsteady concerns (*a*_2_)	0.24	0.044	0.024	− 0.004	0.091
Parental threatening behaviors ➔ Mental incapacitation concerns (*a*_3_)	0.31^**^	0.057	0.016	0.024	0.089
Parental threatening behaviors ➔ Social concerns (*a*_4_)	0.06	0.020	0.018	− 0.016	0.056
Disease concerns ➔ Anxiety (*b*_1_)		0.894	0.393	0.099	1.69
Unsteady concerns ➔ Anxiety (*b*_2_)		0.436	0.613	− 0.801	1.67
Mental incapacitation concerns ➔ Anxiety (*b*_3_)		0.237	0.899	-1.58	2.05
Social concerns ➔ Anxiety (*b*_4_)		0.122	0.680	-1.25	1.50
Parental threatening behaviors ➔ Anxiety (*c*)	0.22^**^	0.233	0.085	− 0.063	0.403
Parental threatening behaviors ➔ Anxiety (*c’*)		0.078	0.084	− 0.093	0.248
Parental threatening behaviors ➔ Disease concerns ➔ Anxiety (*a*_1_*b_1_)		0.120	0.070	− 0.002	0.269
Parental threatening behaviors ➔ Unsteady concerns ➔ Anxiety (*a*_1_*b_2_)		0.019	0.041	− 0.068	0.105
Parental threatening behaviors ➔ Mental incapacitation ➔Anxiety (*a*_3_*b_3_)		0.013	0.062	− 0.127	0.121
Parental threatening behaviors ➔ Social concerns ➔Anxiety (*a*_4_*b_4_)		0.003	0.019	− 0.037	0.045
Parental threatening behaviors ➔ Total anxiety sensitivity ➔ Anxiety (*a*_1,2,3,4_*b_1,2,3,4_)		0.155	0.072	0.007	0.294

Note. *p < .05, **p < .01. The standard error and 95% CI for *a*_n_*b_n_ is obtained via bootstrapping with 10,000 resamples

Parental threatening behaviors (PTI total score) is the predictor and Anxiety (RCMAS total score) is the outcome

Disease concerns, unsteady concerns, mental incapacitation concerns, and social concerns (CASI subscales) are parallel mediators in this model

“Total anxiety sensitivity” is the total indirect effect of all parallel mediators

Path *a*_n_ indicates effect of X on M; *b*_n_, effect of M on Y; *c*, effect of X on Y; *c’*, direct effect of X on Y, controlling for M

Total, direct, and indirect paths are noted after controlling for the variance accounted for by relevant sociodemographic covariates, including age, sex, and race

## Discussion

This investigation examined the role of anxiety sensitivity in the association between parental threatening behaviors and anxiety symptom severity in a sample of trauma-exposed adolescents in acute inpatient care. *Post hoc* exploratory analyses were conducted to explore the facets of anxiety sensitivity in this association. Consistent with our first hypothesis, parental threatening behaviors were significantly and positively associated with anxiety sensitivity. These findings align with prior work documenting associations between harsh parenting and elevated anxiety sensitivity in community samples of adolescents and young adults (Erozkan, 2012; Scher & Stein, 2003; Viana & Stevens, 2016). They further suggest that parental threatening behaviors, in particular, may be associated with interpreting bodily arousal as a sign of danger (Ehlers & Clark, 2000; McLaughlin et al., 2020), which may in turn be associated with increased vulnerability to anxiety (Lei et al., 2023; Scher et al., 2002).

In line with our second hypothesis, greater anxiety sensitivity was associated with more severe anxiety symptoms. This finding aligns with existing cognitive (Clark & Beck, 2010) and expectancy models (Reiss, 1991) of anxiety, as well as prior work in both community and clinical samples of youth (Joiner et al., 2002; Knapp et al., 2016; Silverman et al., 1991), including trauma-exposed samples (Martin et al., 2014). More broadly, findings suggest that catastrophic interpretations of interoceptive sensations can serve to exacerbate and maintain anxiety symptoms—a notable concern given trauma-exposed youths’ heightened susceptibility to negative cognitive appraisals (Claxton et al., 2025; Ehlers et al., 2003; Leen-Feldner et al., 2008).

In line with our fourth hypothesis, tests of indirect effects showed that parental threatening behaviors were indirectly associated with anxiety symptom severity through overall levels of anxiety sensitivity. Although the cross-sectional design precludes inferences about the directionality of these relationships, prior research underscores the profound effects of trauma exposure on the propensity to catastrophize or misinterpret cues (Foa & Kozak, 1986; Joshi et al., 2023; McLaughlin et al., 2019; McLaughlin & Hatzenbuehler, 2009). Such cognitive disturbances also figure prominently in developmental and cognitive models of fear learning (Waters & Craske, 2016), particularly in the context of trauma-exposure (Joshi et al., 2023; Machlin et al., 2024; McLaughlin et al., 2019). From this perspective, it is theoretically plausible that trauma-exposed adolescents who experience parental threatening behaviors may develop cognitive perturbations that generalize to include negative interpretations of interoceptive stimuli (i.e., anxiety sensitivity), thereby increasing anxiety symptom severity.

Interestingly, inconsistent with our third hypothesis, the direct path between parental threatening behavior and anxiety symptom severity was not statistically significant, suggesting that anxiety sensitivity fully statistically accounted for the association between parental threatening behaviors and anxiety symptom severity. This is notable, given past work showing that anxiety sensitivity only partially accounted for the (negative) association between parent emotional availability and adolescent anxiety symptom severity (Kramer & Francis, 2025). Thus, while parental threatening behaviors may influence adolescent anxiety indirectly through anxiety sensitivity, parental emotional availability may serve as an important protective factor, even in the context of high anxiety sensitivity.

Exploratory analyses were conducted to evaluate the roles of the four facets of anxiety sensitivity in the association between parental threatening behaviors and anxiety symptom severity. These analyses revealed that parental threatening behaviors were positively associated with both disease concerns and mental incapacitation concerns, but not with unsteady concerns or social concerns. One potential explanation for this association is that parental threatening behaviors, such as threats of punishment of abandonment, may heighten physiological arousal (La Buissonnière-Ariza et al., 2019), as these behaviors may signal caregiver unavailability. In this context, bodily sensations that typically require parental reassurance (e.g., feeling sick) may instead be experienced as threatening, increasing the likelihood that youth interpret these interoceptive cues as signs of danger (i.e., disease concerns). The association between parental threatening behaviors and the mental incapacitation concerns facet may be partially explained by the trauma-exposed nature of the sample. Indeed, mental incapacitation (e.g., concentration difficulties) symptoms are especially heightened after trauma exposure (Cruz et al., 2022), and may thus be particularly salient in this sample. It is also possible that parental threatening behaviors create uncertainty and fear, leading to feelings of uncontrollability and mental perturbation. However, post-hoc analyses revealed that the indirect association between parental threatening behaviors and anxiety symptom severity was driven by the cumulative effect of the anxiety sensitivity rather than by any facet alone. Additional research is warranted to further explicate potential pathways by which these associations emerge.

The present findings should be interpreted in the context of several limitations. First, although our sample has notable strengths (i.e., diverse sample of trauma-exposed adolescents in acute inpatient care), its size was modest, which limits the stability and precision of the indirect effects. Therefore, results should be interpreted with caution. Relatedly, mean imputation was used for two participants with missing PTI data. Although this approach could theoretically bias estimates, missingness was low (4% of the data), making any effect on the findings likely minimal. Nevertheless, replication with a larger sample would bolster confidence in the significance of our results. Additionally, the use of self-report measures may have contributed to reporting biases and shared method variance, even though self-report measures are especially useful when assessing youths’ internal experiences, which are often not accurately capture by parent or teacher reports (De Los Reyes & Kazdin, 2005). Similarly, parents are also likely to underreport on their own use of harsh or negative parenting behaviors (Korelitz & Garber, 2016), making adolescents a potentially more accurate source. Nevertheless, future studies would benefit from a multi-method, multi-informant approach to examine our theorized model. For example, given evidence of varying effects of maternal and paternal parenting styles on child psychopathology (Pan et al., 2024), future studies should consider assessing both maternal and paternal threatening behaviors. Furthermore, the cross-sectional nature of the design prevents the drawing of causal inferences. Although the sequential ordering of variables in our model is consistent with past work on the development of anxiety sensitivity and anxiety pathology (Lei et al., 2023; Scher & Stein, 2003), it is also possible that there are bidirectional associations between parental threatening behaviors and anxiety sensitivity, wherein expressing fears of interoceptive sensations may elicit parental threats. Additionally, while prospective studies have identified anxiety sensitivity as a predictor of anxiety symptom severity (Allan et al., 2014, 2016; Hernandez Rodriguez et al., 2020; Schmidt et al., 2010), bidirectional associations have been identified as well (Zavos et al., 2012). A future longitudinal design relying on more dynamic assessments (e.g., ecological momentary assessment) would be better suited to model these associations. Finally, the internal consistency of some of the CASI subscales (i.e., mental incapacitation and social concerns) was low. Notably, these subscales contain relatively few items, which can attenuate alpha coefficients. Although the analyses with the CASI subscales were exploratory, future work should consider the internal reliability of the CASI facets.

Despite these limitations, the present study retains important clinical implications. First, the findings underscore the importance of assessing both parental threatening behaviors and anxiety sensitivity among psychiatric inpatient youth. Existing measures, such as the Parent Threat Inventory (Scher et al., 2002) and the Childhood Anxiety Sensitivity Index (Silverman et al., 1991, 2003) are free, relatively brief, and can provide important information for treatment planning purposes. Second, the findings suggest that additional work examining anxiety sensitivity as an intervention target among youth in inpatient psychiatric care may be useful —particularly those with histories of problematic parenting. Interventions targeting anxiety sensitivity have shown promise among adolescent populations, demonstrating reductions in anxiety symptoms and improved coping with physiological arousal (García-Lorenzo et al., 2025; Knapp et al., 2020; Schiele et al., 2021). Moreover, these interventions are brief and easily implementable (e.g., computer-delivered; El Jamal et al., 2025) in a psychiatric inpatient setting, where time and space constraints are usually high (Gupta et al., 2024; Raphael et al., 2021). Finally, results can inform future work exploring the role of other cognitive mechanisms (e.g., interpretation biases) in anxiety psychopathology among trauma-exposed youth.

Overall, the present study found that greater exposure to parental threatening behaviors was significantly associated with more severe anxiety sensitivity and anxiety symptoms in a sample of trauma-exposed adolescents in psychiatric inpatient care. Additionally, anxiety sensitivity statistically mediated the association between parental threatening behaviors and anxiety symptom severity. Findings underscore the association of parental threatening behaviors with anxiety psychopathology in this population, as well as the potential utility of further evaluating anxiety sensitivity-focused interventions for this population (Fitzgerald et al., 2021; Knapp et al., 2020).

## Data Availability

No datasets were generated during the current study. Data are available upon reasonable request.

## Declarations

### AI Statement

No artificial intelligence (AI) tools were used in the conduct of the research or preparation of the manuscript.
